# Physical Activity in Immersive Virtual Reality: A Scoping Review

**DOI:** 10.3390/healthcare11111553

**Published:** 2023-05-25

**Authors:** Frano Giakoni-Ramírez, Andrés Godoy-Cumillaf, Sebastián Espoz-Lazo, Daniel Duclos-Bastias, Pablo del Val Martín

**Affiliations:** 1 Faculty of Education and Social Sciences, Universidad Andres Bello, Las Condes, Santiago 7550000, Chile; 2 Grupo de Investigación en Educación Física, Salud y Calidad de Vida, Facultad de Educación, Universidad Autónoma de Chile, Temuco 4780000, Chile; 3 Facultad de Ciencias Para el Cuidado de la Salud, Universidad San Sebastián, Sede Los Leones 5090660, Chile; 4 Escuela de Educación Física, Pontificia Universidad Católica de Valparaíso, Valparaíso 2374631, Chile; 5 IGOID Research Group, Physical Activity and Sport Science Department, University of Castilla-La Mancha, 45071 Toledo, Spain

**Keywords:** digital technology, health, innovation, exercise, technologic gadget

## Abstract

Physical activity has benefits for health, but many adolescents are inactive. However, video games such as Immersive Virtual Reality (IVR) have grown in popularity as a leisure activity among young people, allowing them to manipulate objects in virtual environments increasing the practice of physical activity. The evidence indicates that the interest in physical activity through IVR is greater than in traditional methods, and different experiences have been reported. However, few studies indicate the sample evaluated, the effects found, or the IVR instruments used. Due to this, is the aim of this study is to identify the publications referring to IVR and physical activity, characterize them, and present the obtained main findings. For this, the guidelines described in the PRISMA-ScR for scoping reviews were applied. After the use of the inclusion and exclusion criteria, eight articles were included. Results show evidence regarding physiological outcomes, perceptual variables, interest and enjoyment, and psychological effects regarding physical activity through IVR. Additionally, the use of different devices and their prescriptions are explored. It is concluded that there is interest from the scientific community for the practice of physical activity through IVR, as well as for its application for the maintenance of active habits. This is important as it positions IVR as a method that can be a more experiential and effective way to develop and maintain a healthy lifestyle.

## 1. Introduction

In recent years, the effects produced by physical activity (PA) on the population’s health have been highly documented [1,2]. However, regardless of the positive benefits, around 20% of adults and 80% of adolescents worldwide are physically inactive, in part due to social- and daily-life changes [3], especially since COVID-19 restrictions were imposed by governments to avoid their populations contracting the disease. Worryingly, it was precisely in the latter age group where PA levels decreased drastically [4,5]. One of the reasons that might explain this behavior, is that PA is frequently seen as boring and hard, pushing people away from PA-related behaviors, especially after extended days of work [6]. Other factors that affect motivation towards PA practice are beliefs, values, social influences, and family support, as well as the physical and sports habits of the immediate environment, which can generate positive or negative attitudes towards PA [7,8].

Nowadays, it can be seen that many individuals are interested in practicing actions involving video games during their leisure activities, since through these, entertainment can be obtained as well as relaxing time during the same task [6]. This phenomenon is not recent and started to increase in popularity among the young population around the beginning of the 2000s [9]. Even though investigation into video games has largely focused on their problematic use and the addiction they cause [10,11,12], the evolution they have undergone regarding integrating PA has aroused interest due to the fact that they could contribute to improving PA behavior. In this sense, Freina and Ott [13] concluded in their work that the motivation to use this kind of technology is that it allows users to experience situations that in reality would be unfeasible, particularly due to time and/or space, or due to the high level of harm users may experience by performing the same activities in real life.

Currently, the technological tool Virtual Reality (VR) has gained importance. This digital instrument creates artificial sensory experiences in which visual, auditory, tactile, and olfactory stimuli let users handle objects within a virtual environment, such as forests, cities or gyms, recreating scenarios that allow the practice of PA by helping to induce a sense of mental or physical presence [14,15]. Evidence affirms that the interest in performing PA through virtual reality is higher compared to similar manifestations of PA in traditional approaches [14,15,16].

Among the different varieties of VR is immersive (IVR) which is based on the use of head-mounted displays (head mountain display), body motion sensors, advanced interface devices (e.g., dedicated headsets), and real-time graphics to mimic a completely virtual environment for operators [17,18], in which perceptions of time and the real world tend to be disconnected [19].

In recent years, the practice of PA through IVR has been identified as a new approach to encourage PA and healthy behaviors, being increasingly used in health promotion [20] as it delivers psychological benefits and increases the likelihood of long-term exercise adherence [21,22].

Due to the technological advances achieved, VR has attracted the attention of various fields related to public health, through the integration of exercise and rehabilitation equipment that has been used to promote the practice of PA, thus offering the chance to strengthen repetitive tasks and increase graphic and acoustic feedback, which makes IVR more stimulating than traditional VR, without posing a significant risk or being limiting for participants [23].

Although the scientific evidence available to date provides arguments that support IVR as an instrument that affords health benefits, the following research questions arise: what are the useful applications of IVR? What are the characteristics of the studies that have used this technology? What scope has it had in terms of its application? For these reasons, a scoping review will be carried out to systematically map the research carried out in this area, as well as to identify existing gaps in knowledge. For these reasons, this scoping review aims to (1) identify the publications referring to IVR and physical activity, (2) identify the IVR instruments and materials used, (3) identify the instruments used to measure the amount of physical activity performed using IVR, and (4) present the main findings obtained.

## 2. Materials and Methods

This scoping review was reported following the PRISMA extension for scoping review (PRISMA-ScR): Checklist and explanations [24] (Supplementary material).

A bibliographic review in the Web of Science and Scopus databases was carried out by two independent researchers. The selected articles were those that evidenced the application of IVR in the field of physical activity. To be incorporated, articles had to meet the following inclusion criteria: (a) Have used VR equipment; (b) involved human subjects; (c) involved some type of physical activity (dancing, playing a sport, walking, etc.); and (d) published in English until December 2021. In addition to the above, articles that met the following exclusion criteria were discarded: (a) included self-reported data of what was conducted with VR; (b) the use of non-immersive VR instrumentation; and (c) with findings not related to physiological, and/or psychological outcomes. Bibliographic searches were restricted to keywords, titles and abstracts. Search terms were combined using the Boolean OR operator and searched simultaneously with other search groups based on the PICO elements (population, intervention, comparison, outcome, and study design) using the Boolean AND operator (Table 1). To find root words, proximity operators (“*”) were used. The search strategy used was based on the terms described in Table 1.

Once duplicate studies were excluded, two investigators evaluated the titles and abstracts of included articles to recognize eligible studies. Abstracts that partly met the inclusion/exclusion criteria (i.e., did not provide sufficient information) were assessed by reading the full text. Afterwards, the same two investigators reviewed the included and excluded articles to confirm the reason for every decision. A third investigator made the final decision by examining and solving discrepancies between the two investigators, based firmly on the inclusion/exclusion criteria.

Regarding the data extraction, the subsequent information was obtained: (1) year of publication; (2) country; (3) study design (i.e., sample characteristics, research duration, VR exposure, outcomes associated with physiological, rehabilitation, and/or psychological results, and instruments used); and (4) significant findings on the efficacy of VR on products related to the above results.

**Figure 1:**
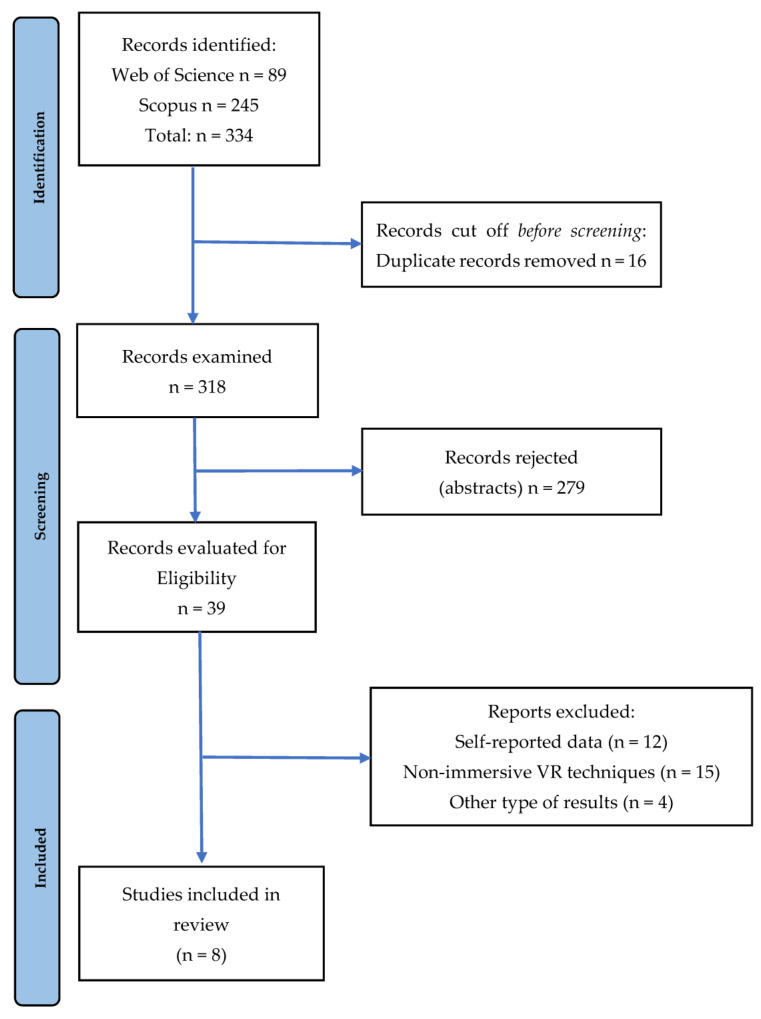
Flow diagram of the studies through the revision process.

The flow diagram of the scoping review presented in Figure 1 describes the review process carried out. Initially, a total of 334 articles were found, of which 16 duplicates were discarded. Of the remaining 318 articles, the abstracts were read and 299 that did not meet the criteria of inclusion were discarded. Of the 19 resulting articles, 31 were discarded as they met one of the exclusion criteria: reason 1: self-reported data (*n* = 12), reason 2: non-immersive VR techniques (*n* = 15), and reason 3: another type of result (*n* = 4). Once the review work was completed, the articles that met the criteria for inclusion in the present study were selected (*n* = 8), all with a cross-sectional design.

## 3. Results

The selected investigations were conducted between 2019 and 2021 and took place in different countries: Poland [25,26,27], France [28], USA [29], Germany [30], Israel [31] and the UK [16]. The sample sizes ranged from 4 to 80 subjects and the age of the participants comprised a range between 10,1 and 81,2 years, among which most of the articles targeted an adult population; however, one article had a sample of child participants [25] and another with older adults [30] (Table 2). The intervention times varied by study, as distinct investigations examined numerous variables.

In some studies [25,26,27,30,32], the most commonly used IVR devices were the HTC Vive^®^ and the specific games used varied depending on the different investigation objectives. For example, VirZomm VR^®^ was used during interventions where the simulation of a bicycle ride was the main focus. Regarding the study setting, some of them were conducted in a highly controlled laboratory [16,27,28,29], while the rest did not specifically report the location in which the study was conducted [25,26,30,32].

The measurement techniques in the included articles were legitimate measurement tools and the data collection process was conducted by skilled personnel. Taking into account the physiological variables, heart rate was evaluated through a POLAR^®^ brand heart rate monitor device [16,27,30] and by the Vantage V^®^ heart rate monitor [25,26], being the most common measurement tools; to a lesser extent the E4 Biosensor Wristband^®^ that also records electrodermal activity [32] and the electrocardiogram (X12+, Mortara) [28] were used. On the other hand, the most commonly used equipment for physical activity was the Virtuix Omni^®^ [25,26,32], while the less-used pieces of equipment for this purpose were the ICAROS Pro Flight Simulator^®^ [26] together with the Spirit Fitness^®^ (156 XBU55 Upright Bike) [29].

Regarding perceptual variables, one article used the MEC Spatial Presence Questionnaire (MEC—SPQ) to measure attention, involvement and spatial presence [30]. Regarding the immersive experience of the participants, one study used the Immersive Experience Questionnaire [16].

To assess inherent interest and enjoyment when executing a specific activity, two studies used the Interest Enjoyment subscale of the Intrinsic Motivation Inventory (IMI) [26,30]. On the other hand, the Situational Motivation Questionnaire (SM) was used in one study to measure motivation [29]. In relation to mental demand, physical demand, temporal demand, own performance, effort and frustration, the NASA-TLX Questionnaire was applied in one article [30].

Among the documents included in this review, four [16,26,29,30] of the eight articles that evaluated the effect of IVR exercise on psychological variables showed positive effects. However, these studies demonstrated that physical exercise performed with IVR generates an above-average perceived exertion (6.8 points) [25] and a high enjoyment ratio (5.74 points) [26], and is effective in reducing pain [16]. Moreover, the cognitive aspect of physical exercises in immersive virtual reality can generate the sensation of physical load, even if simple movements are achieved. Finally, concerning motivation, IVR was found to generate more intrinsic motivation (6.31) and identified regulation (6.03) than non-immersive VR and traditional cycling [29].

For its part, the efficacy of IVR on PA outcomes was measured mainly by heart rate [16,25,26,27,28,30,32]. In the studies using Omni Treadmill^®^ and Icaros Pro Flight ^®^ it was observed that heart rate was 155.5 bpm–167.0 bpm [25] and 121.36 bpm–149.55 bpm [26], respectively, reaching a high-intensity PA with an average of 76.8% HRmax. The study using IVR with a heart rate monitor in an electronically braked cycle ergometer obtained an average HR of 158.7 bpm [27].

With lower results than those previously mentioned, the remaining studies recorded average HRs of 95.67 bpm [32], and 87.35 bpm [16] respectively.

## 4. Discussion

The present review aimed to identify the limit to which IVR has been used in the area of physical activity and to describe the findings that have been obtained. This study is the first to review articles that have used IVR in the field of physical activity. The findings show that there are scientific interests in advancing the generation of knowledge around IVR. This was verified by the volume of papers that were identified in the first survey conducted, emphasizing the ability of PA using IVR as a modern trend in the healthcare field [15,33,34] and allowing us to showcase the high rate of publication of articles regarding the use and applications of this digital tool.

Along with the above, this review allows us to establish that experiences using IVR in the promotion of physical activity have important stimulation effects on heart rate at different levels of intensity, so that the use of IVR at moderate- and vigorous-intensity activity could contribute to obtaining the benefits that the WHO describes after achieving weekly recommendations of physical activity volume and intensity [35].

On the other hand, the innovative and novel characteristics of the PA experience in IVR contribute to increasing PA pleasurable experience in the subjects, even boosting the levels of intrinsic motivation. This could have a positive effect on the decrease in the perception of pain associated with exercise [16], calling attention to the usefulness of IVR in subjects evaluated with these training devices. Additionally, the research reviewed supports the use of IVR since it may have a high potential to increase adherence to physical activity, which is an important issue in counteracting insufficient physical activity, regularly highlighted in research as the main reason for not following health guidelines. This situation goes hand in hand with previous evidence which indicates that the regular practice of physical activity in IVR has beneficial effects on health in various populations [14,15,16,20,21,22]. Likewise, the use of virtual reality can improve health, knowledge, motor skills and complex problem-solving techniques in different populations [36].

Future research must utilize high-quality designs along with diverse potential populations (e.g., with specific characteristics) [37]. In addition, the involvement duration of future experiments should be extended, as well as post-intervention follow-ups to ensure the long-term efficacy of the interventions. Moreover, potential confounders such as age, gender, and socioeconomic status should as well be taken into account. Finally, different experiences of IVR may produce distinct effects and should therefore be meticulously analyzed in different contexts. However, based on our review, it can be noted that IVR is safe and has the capacity to improve physiological, and psychological outcomes after being well implemented by trained practitioners [29].

### 4.1. Practical Implications

The results of this review have functional implications for investigators, physical activity, and health professionals. These technological resources can be used on a large scale because they have been used in different fields of research for more than a decade [38,39,40]. In addition, IVR was proven to be profitable and safe, which is why new and innovative immersive technologies are being created and used in training [41]. Indeed, the appropriate use of IVR may be a higher experiential and successful approach to developing and sustaining a healthy lifestyle. Given the prospective negative effects of IVR devices (e.g., motion sickness), other equipment, such as non-immersive or interactive, can be chosen based on reality. In addition, the cost of IVR equipment is high and should be taken into account. Customers can select a lightweight and simple device or use apps on their phone that are like IVR devices, while gyms or fitness centers can choose greater value and multifunction IVR tools to take on the needs of target populations.

### 4.2. Limitations

Some limitations of this study should be noted that may have compromised its results. Firstly, only articles published in English were involved, which might ignore relevant research available in other languages; secondly, due to the inclusion criteria, a limited number of articles were incorporated; thirdly, a scarcity of included studies had based their protocols on very controlled laboratory locations, which could limit the external validity of the results, and some studies omitted to report on their experimental setting. It is also important to note that IVR technology is not yet a piece of fully mature equipment and it has numerous limitations. Primarily, these include generating a high-fidelity simulation remains a thought-provoking task, so IVR may reduce the sense of presence, leading to limited realism of humanoid and automobile behavior and motion; a limited level of interaction among users and IVR; imperfection in treadmill walking mechanisms; and the relatively low display resolution and field of view (in HMD). Therefore, it is prudent to include indicators that directly analyze the consequences of these limitations. Secondly, the employment of IVR requires more supplies compared to conventional surveys and experimental methods [31,42,43]. Thirdly, the use of IVR devices in general and walking emulators particularly still demands some professional expertise, which is likely to be an obstacle to implementation. Fourthly, an IVR research session is a lengthy procedure, which may limit the amount of participants. In addition, the number of assessments that each participant can experience is also restrictd, as it is prudent to restrict the duration of the sessions. In addition, studies are often limited to participant investigations in novel settings. It is important to note that many of the above limitations are expected to be resolved with the advancement of technology and its constant adaptation for research reasons.

## 5. Conclusions

This scoping review identified studies that show the contribution of IVR in the study of motor function, psychological effects, and heart rate intensity during physical activity. Although IVR is an area that has been little investigated, the results indicate that there is interest from the scientific community in its research, and its application to physical activity is a current trend in the development and maintenance of active habits through heart rate stimulation, contributing to international recommendations. Together with the above, research on the use of IVR suggests effects on the motivation of participants, supporting IVR as a method that can be a more experiential and effective way to develop and maintain a healthy lifestyle.

## Figures and Tables

**Table 1:** Search Strategy.

PICO Components	Keywords
#1 Population	“virtual reality” OR “virtual environment” OR “immersive virtual environment” OR immersion OR “active gaming” OR “immersive virtual reality” OR “VR” OR “IVR”
#2 Intervention	exercise OR “physical activity” OR training OR “active gaming” OR “video games” OR “video exercise” OR “virtual high-intensity training”
#3 Outcome	“health-oriented physical activity” OR “heart rate” OR “intensity of physical activity” OR “energy expenditure”
#4 Study design	“randomized controlled trial” OR RCT OR “non-randomized clinical trial” OR “pre-post study” OR “controlled trial” OR “clinical trial” OR intervention OR random *
Search strategy	#1 AND #2 AND #3 AND #4

**Table 2:** Studies included in the scoping review.

		Journal	Country	Aim	Sample (*n*)	Age (X ± SD)	Physical Activity Measure	Instruments and Materials	Key Findings
1	[32]	International Journal of Environmental Research and Public Health	Israel	Evaluate the capabilities and opportunities the technology offers, and test the feasibility of implementing a VR walking simulator for research purposes with human participants.	*n* = 4 2 men 2 women	37.5 ± 15	Electrodermal activity Heart rate Number of steps Cadence (steps/min) Step regularity Step symmetry	HTC Vive Pro Eye Virtuix Omni E4 biosensor wristband	EDA levels ranged between 4.435 µS to 28.401 µS, and heart rates (HR) ranged between 82 and 115 bpm.
2	[25]	International Journal of Environmental Research and Public Health	Poland	Assess the attractiveness and intensity of physical exercise of obese children while playing active video games (AVGs) in IVR on an omnidirectional treadmill, and present the results compared to health recommendations (PA).	*n* = 11 7 boys 4 girls	10.1 ± 1.7	Heart rate Perceived exertion	HTC Vive Virtuix Omni Vantage V heart rate monitor	The intensity of physical activity while playing two games was high (HRavg > 77% HRmax). The perceived exertion was rated at 6.8 points.
3	[26]	International Journal of Environmental Research and Public Health	Poland	Assess the enjoyment and intensity of physical exercise while practicing physical activity (PA) in immersive virtual reality (IVR) using innovative training devices.	*n* = 61 51 men 10 women	26.0 ± 8.8	Heart rate Interest Enjoyment	HTC Vive Virtuix Omni Icaros Pro flight simulator Vantage V heart rate monitor Intrinsic Motivation Inventory (IMI)	High enjoyment rating during physical activity in VR (5.74 points). The heart rate was 149.55 bpm. The intensity of physical activity during games on training devices was at the level recommended for health benefits for 80.55% of its duration
4	[16]	Psychology of Sport & Exercise	UK	Investigate whether the effectiveness of VR in reducing the feeling of exercise pain and effort is moderated by PBC.	*n* = 80 21 men 59 women	23.0 ± 5.0	Heart rate Time of exhaustion Pain intensity rating Rating of perceived Exertion Private body consciousness Immersive experience	Samsung Gear VR Polar electro N2965 Cook Scale Borg Scale Immersive Experience Questionnaire	Virtual reality was effective in reducing exercise pain. Participants who had exercised in VR had a lower HR (~3 bpm lower) than participants who had exercised outside VR.
5	[30]	Societies	Germany	Assess a VR exergame that features rhythmic movements in 3D space and compare this to a traditional 2D gymnastics video	*n* = 25 older adults 3 men 22 women	81.2 ± 4.97	Heart rate Interest Enjoyment Well-being Attention allocation Perceived workload	Simulator Sickness Questionnaire (SSQ) MEC Spatial Presence Questionnaire (MEC-SPQ) Intrinsic Motivation Inventory (IMI) NASA-TLX Questionnaire Polar OH1 heart rate sensor Valve Index VR headset	The cognitive aspect of physical exercises in virtual reality can lead to the feeling of a physical burden, even if easy movements are performed. SSQ was 8.98, IMI was 4.57, MEC-SPQ was 4.44, NASA-TLX was 19.77 and heart rate was mean 76.77 bpm.
6	[27]	Frontiers in Physiology	USA	Assess the influence of immersive virtual reality (VR) on exercise tolerance expressed as the duration of a submaximal exercise test (ET) on a cycle ergometer.	*n* = 65 17 men 48 women	23.7 ± 1.0	Heart rate variability	HTC Vive Pro Google HTC Vive Tracker Polar H10 cycle ergometer (Lode Excalibur Sport)	The immersive virtual reality stimulation leads to a reduced heart rate response during a submaximal exercise test, and consequently, to the subject reaching a higher work rate before the target heart rate was achieved.
7	[28]	International Journal of Environmental Research and Public Health	France	Compare the physical load elicited by conventional exercise and AG with an HMD.	*n* = 9 men	27 ± 5	Heart rate Age-predicted maximal HR Relative energy expenditure Oxygen consumption	HTC Vive VR (OPJT100) electrocardiogram (X12+, Mortara) Breath-by-breath indirect calorimetry system	Active gaming in head-mounted displays resulted in a greater psycho-physiological strain without accompanying increases in relative energy expenditure. Could elicit levels of physical activity typically found during conventional exercise.
8	[29]	Journal of Clinical Medicine	USA	Examine differences in young adults’ situational motivation (SM) among immersive VR, non-immersive VR, and traditional stationary cycling sessions	*n* = 49 14 men 35 women	23.6 ± 3.39	Intrinsic Motivation Identified regulation External regulation Amotivation	PlayStation VR VR Headset, Xbox 360 Situacional Motivation Questionnaire (SM) Gamercize Bike Spirit Fitness (156 XBU55 Upright Bike) Tanita BC-558 SECA stadiometer	Immersive VR cycling caused higher intrinsic motivation (6.31) and identified regulation (6.03). Similarly, it had lower external regulation (2.72) and motivation (1.60), compared to non-immersive VR cycling and traditional cycling.

## Data Availability

The data presented in this study are available on request from the corresponding author.

## Supplementary Materials

The following supporting information can be downloaded at: https://www.mdpi.com/article/10.3390/healthcare11111553/s1; S1: Preferred Reporting Items for Systematic reviews and Meta-Analyses extension for Scoping Reviews (PRISMA-ScR) Checklist.
